# Supplementary material for: A tripartite synergistic optimization strategy for zinc-iodine batteries
Source: Nat Commun. 2024 Nov 9;15:9702. doi: 10.1038/s41467-024-53800-6 (PMC11549484; doi:10.1038/s41467-024-53800-6)
Supplement: Supplementary file 1 — Supplementary information [file 41467_2024_53800_MOESM1_ESM.pdf]

Supplementary Information

A tripartite synergistic optimization strategy for zinc-iodine batteries

Weibin Yan[1], Ying Liu[1,2]*, Jiazhen Qiu[1], Feipeng Tan[1], Jiahui Liang[1], Xinze Cai[1], Chunlong Dai[1,2], Jiangqi Zhao[1,2]*, Zifeng Lin[1,2]*

[1] College of Materials Science and Engineering, Sichuan University, Chengdu, China.

[2] Key Laboratory of Advanced Special Material & Technology, Ministry of Education, Chengdu 610065, China.

*email: liuying5536@scu.edu.cn; jiangqizhao@scu.edu.cn; linzifeng@scu.edu.cn

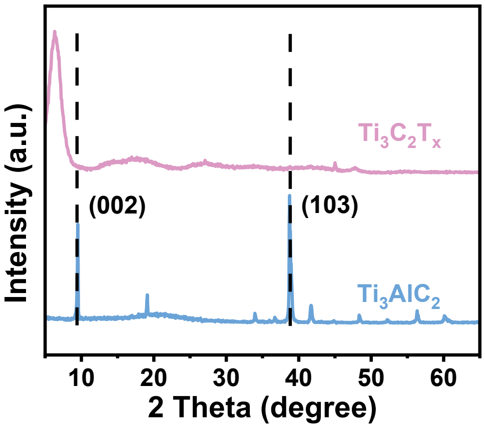

Supplementary Fig. 1 XRD patterns of Ti_3AlC_2 and $Ti_3C_2T_x$ MXene.

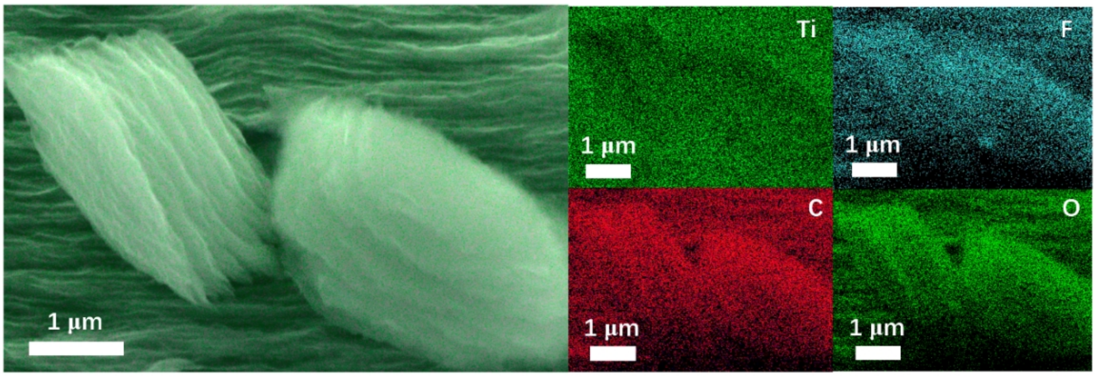

Supplementary Fig. 2 Elemental mapping images of Ti, F, C, and O species.

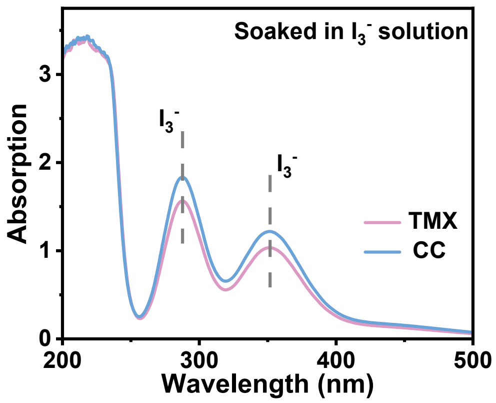

Supplementary Fig. 3 UV-vis spectra of I_3^-/H_2O solutions after TMX and CC soaked for 7 days.

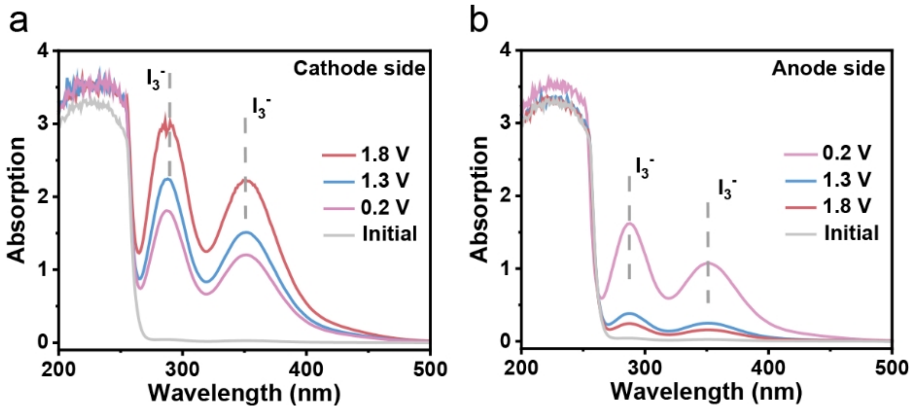

Supplementary Fig. 4 The UV spectrum of ZSI electrolyte at (a) cathode side and (b) anode side under different charge states.

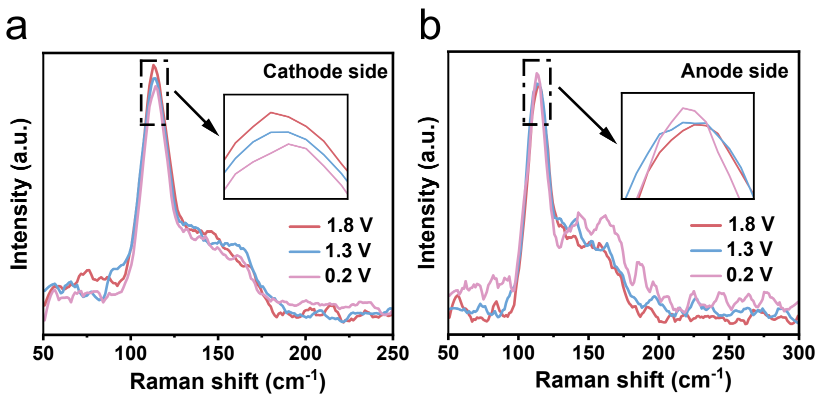

Supplementary Fig. 5 The Raman spectrum of ZSI electrolyte at (a) cathode side and (b) anode side under different charge states (The illustrations are partial enlarged views).

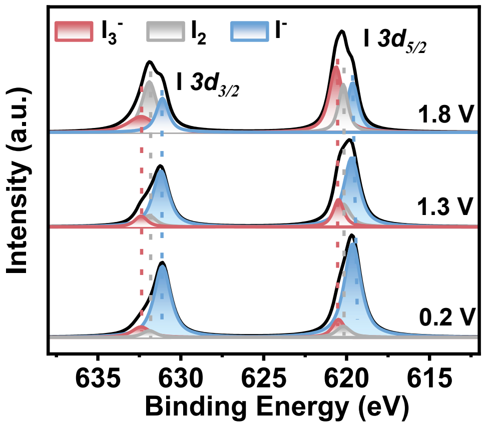

Supplementary Fig. 6 The XPS of cathode electrode under different charge states.

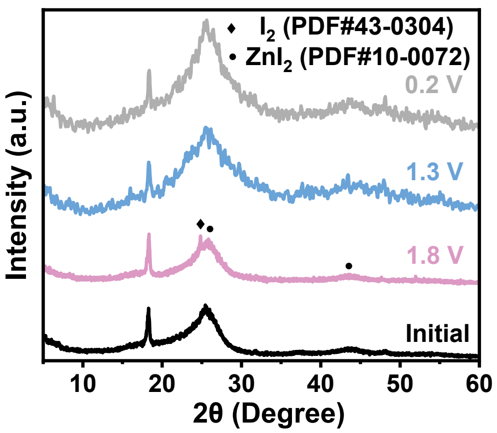

Supplementary Fig. 7 The XRD of cathode electrode under different charge states.

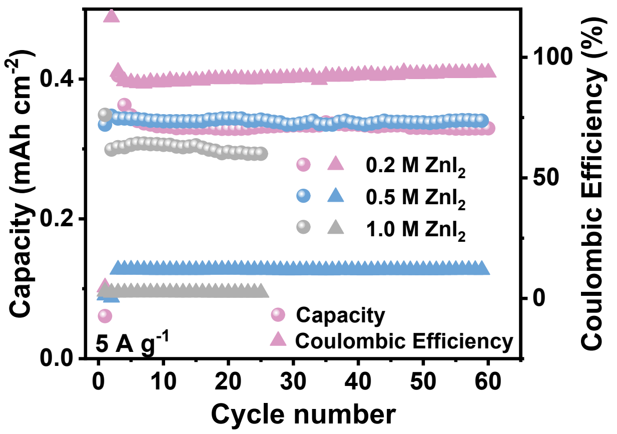

Supplementary Fig. 8 Capacity and Coulombic efficiency of different contents of ZnI_2 (2 M $ZnSO_4$+0.2 M ZnI_2, 2 M $ZnSO_4$+0.5 M ZnI_2 and 2 M $ZnSO_4$+1.0 M ZnI_2)

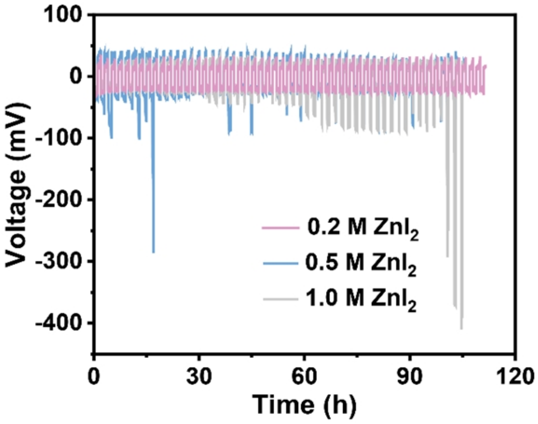

Supplementary Fig. 9 Zn//Zn symmetrical tests with different contents of ZnI_2 (2 M $ZnSO_4$+0.2 M ZnI_2, 2 M $ZnSO_4$+0.5 M ZnI_2 and 2 M $ZnSO_4$+1.0 M ZnI_2)

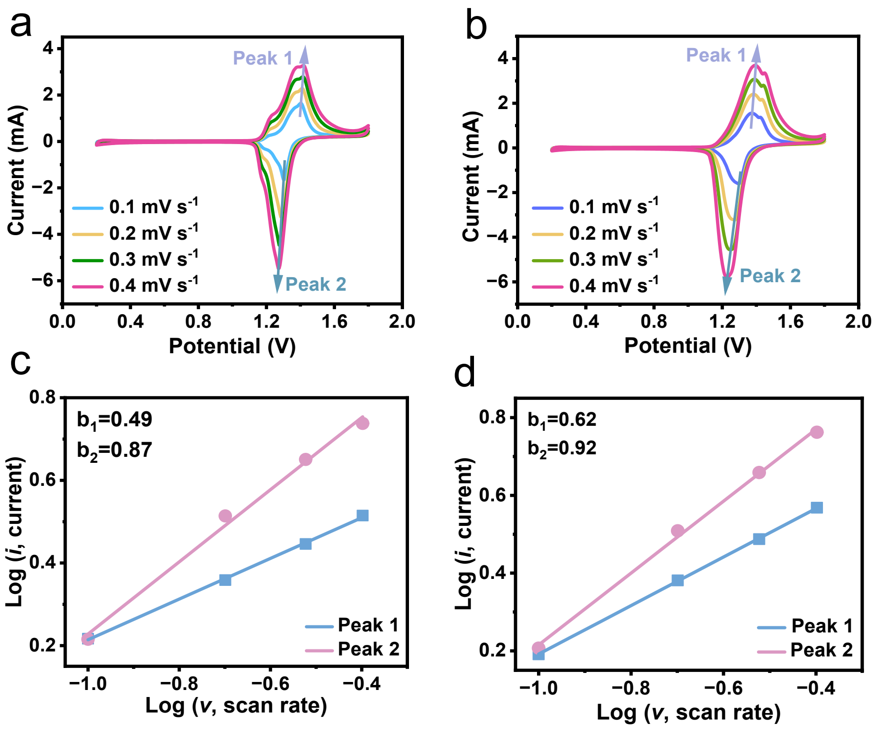

Supplementary Fig. 10 CV profiles of (a) CC and (b) TMX at different scan rates. The fitting lines between log(i) and log(v) at specific peak currents from CV curves: (c) CC and (d) TMX.

The reaction kinetics of CC and TMX were investigated by CV measurements at various scan rates (0.1−0.4 mV s^{-1}) (Supplementary Figs. 10a-10b). As the scan rate increases, the CV curves exhibit similar shapes, with the redox peaks gradually enlarging. The relationship between the peak current (i) and scan rate (v) follows a power law ($I = av^b$, where a and b are adjustable parameters). For CC, the calculated b values for peaks 1 and 2 are 0.49 and 0.87, respectively (Supplementary Fig. 10c), significantly lower than those of TMX (Supplementary Fig. 10d, 0.62 and 0.92, respectively), indicating a more favorable redox kinetics in TMX.

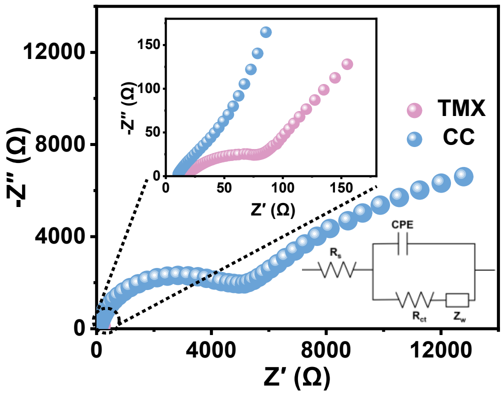

Supplementary Fig. 11 EIS curves of CC and TMX. (The illustration shows the corresponding equivalent circuit)

The electrochemical impedance spectroscopy (EIS) profiles reflect rapid reaction kinetics with smaller charge transfer resistance (R_{ct}: 6965 Ω for CC and 245 Ω for TMX). Moreover, the solution resistance of TMX (R_s=10 Ω) is smaller than that of CC (R_s=11 Ω). Compared to CC, the plot of TMX exhibits a higher slope, indicating a lower ion diffusion resistance.

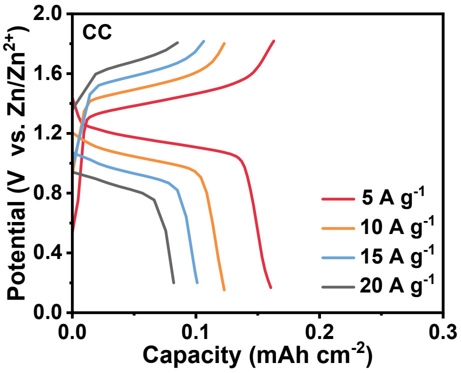

Supplementary Fig. 12 The discharge/charge profiles of CC at different specific current.

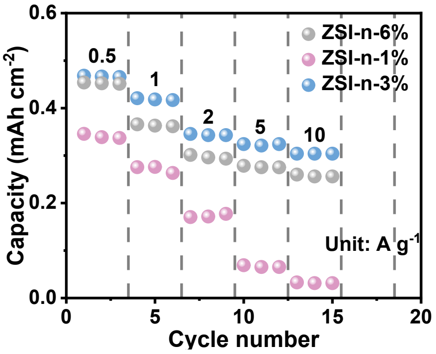

Supplementary Fig. 13 Rate performance comparison of ZSI-*n*-1%, ZSI-*n*-3% and ZSI-*n*-6%.

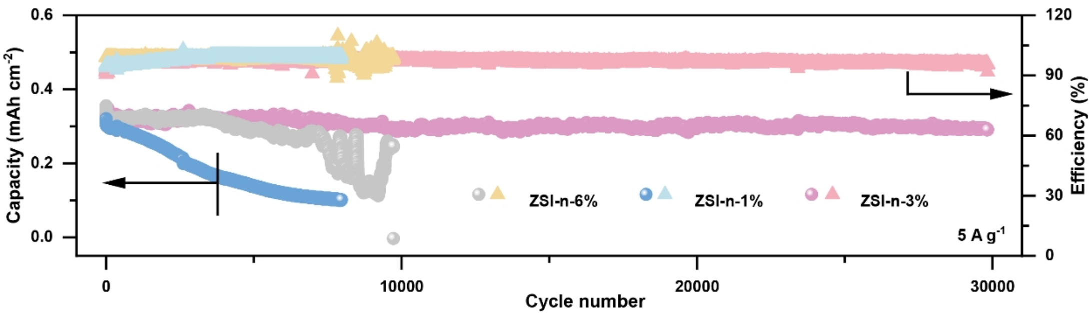

Supplementary Fig. 14 Capacity and Coulombic efficiency of ZSI-*n*-1%, ZSI-*n*-3% and ZSI-*n*-6% at 5 A g^{-1}.

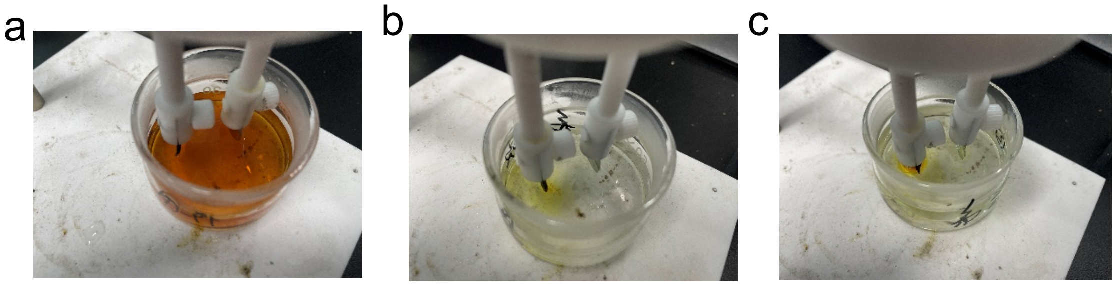

Supplementary Fig. 15 Photos of (a) ZSI-*n*-1%, (b) ZSI-*n*-3% and (c) ZSI-*n*-6% systems in fully charged state.

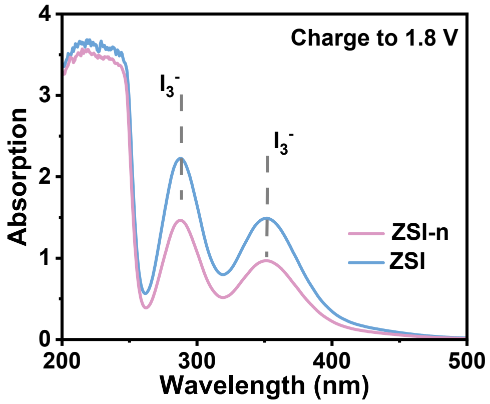

Supplementary Fig. 16 UV-vis spectra of ZSI-*n* and ZSI electrolyte after charging to 1.8 V.

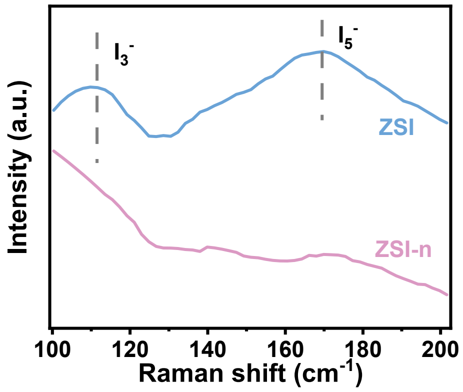

Supplementary Fig. 17 Raman spectra of ZSI-*n* and ZSI electrolyte after charging to 1.8 V.

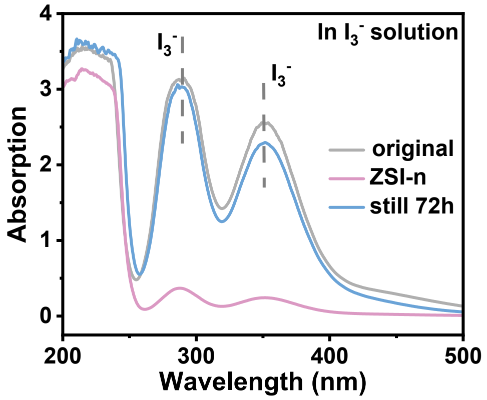

Supplementary Fig. 18 UV-vis spectra of I_3^-/H_2O + ZSI-*n* and I_3^-/H_2O solutions still for 72 h.

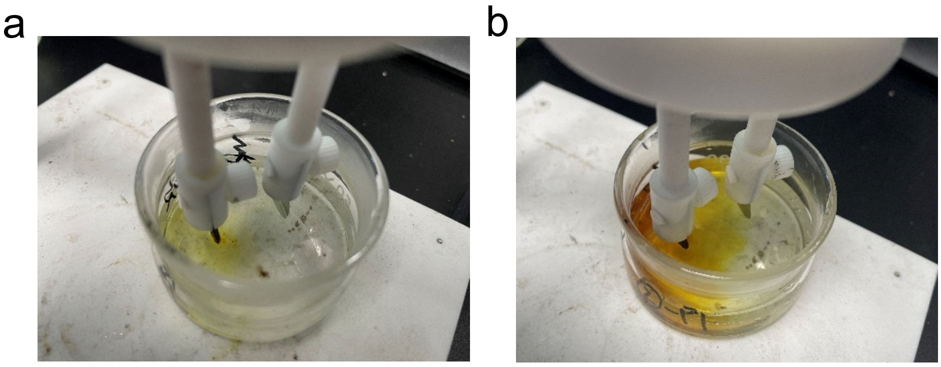

Supplementary Fig. 19 Photos of (a) ZSI-*n* and (b) ZSI systems in fully charged state (The left electrode is cathode and the right electrode is anode in these two photos).

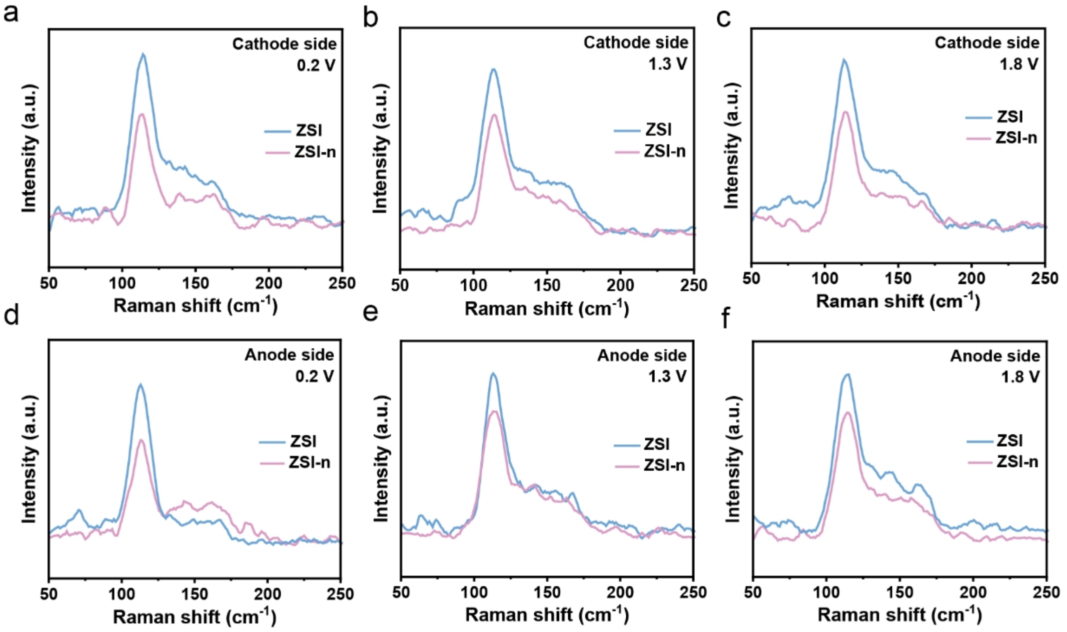

Supplementary Fig. 20 The UV spectrum of ZSI and ZSI-*n* electrolyte at cathode side under (a) 0.2 V, (b) 1.3 V, (c) 1.8 V and anode side under (d) 0.2 V, (e) 1.3 V, (f) 1.8 V.

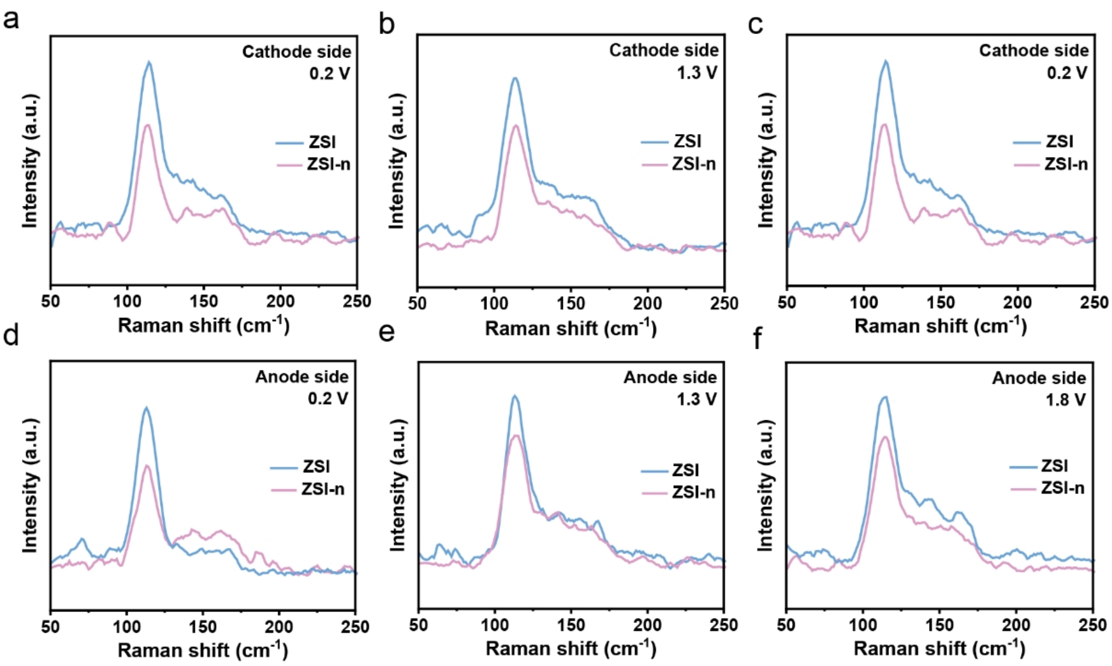

Supplementary Fig. 21 The Raman spectrums of ZSI and ZSI-*n* electrolyte at cathode side under (a) 0.2 V, (b) 1.3 V, (c) 1.8 V and anode side under (d) 0.2 V, (e) 1.3 V, (f) 1.8 V.

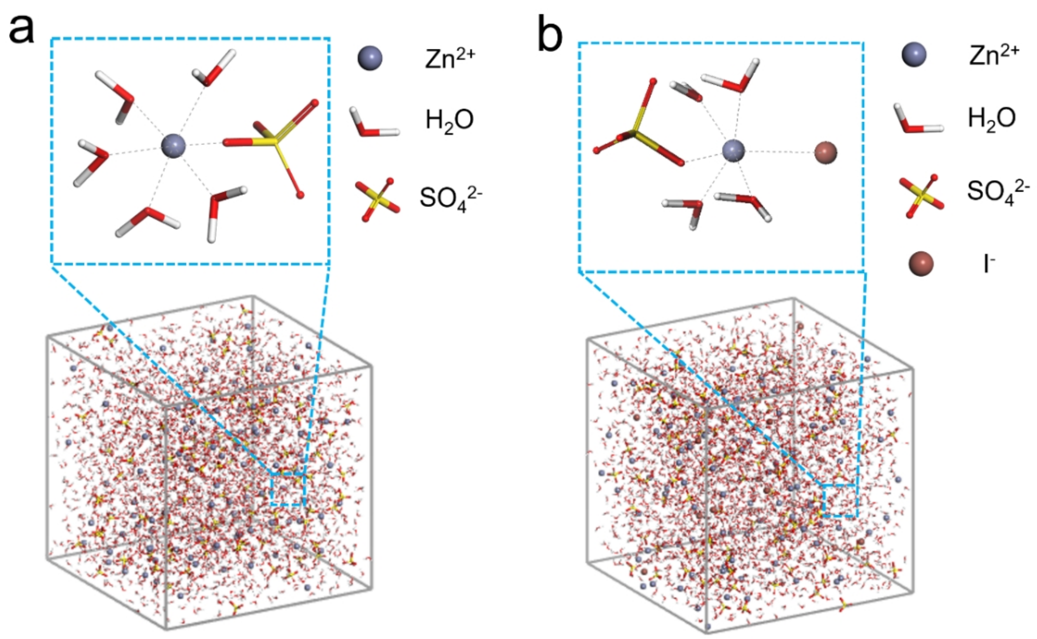

Supplementary Fig. 22 Snapshots of MD simulation boxes for (a) ZS and (b) ZSI electrolyte.

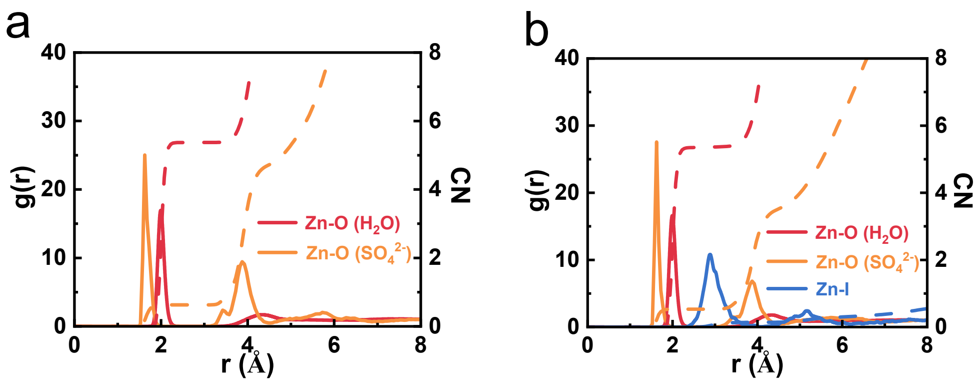

Supplementary Fig. 23 RDFs for Zn^{2+} pairs in (a) ZS and (b) ZSI.

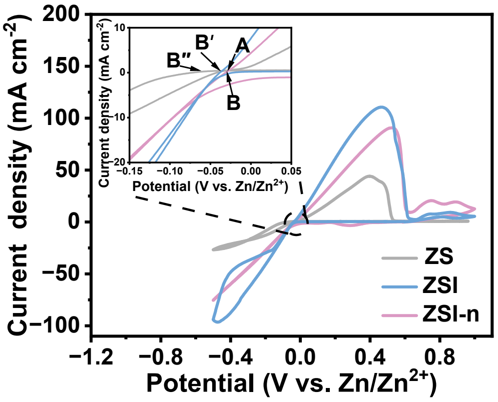

Supplementary Fig. 24 CV curves of Zn//Cu cells in ZS, ZSI and ZSI-*n* electrolytes. LCSM images of Zn anodes after cycling in three electrolytes.

Cyclic voltammetry (CV) tests on Zn//Zn symmetric cells were employed to elucidate the mechanism of the zinc nucleation stage. As shown in Supplementary Fig. 24, the distance between the intersection point (A) and the inflection point at which Zn^{2+} is initially reduced (B/B′/B″) can be considered the nucleation overpotential.[26] The lower nucleation overpotential of 27 and 36 mV in ZSI-*n* and ZSI electrolytes, compared to 67 mV in ZS, indicating the beneficial impact of introducing I^- and *n*-butanol on zinc deposition.

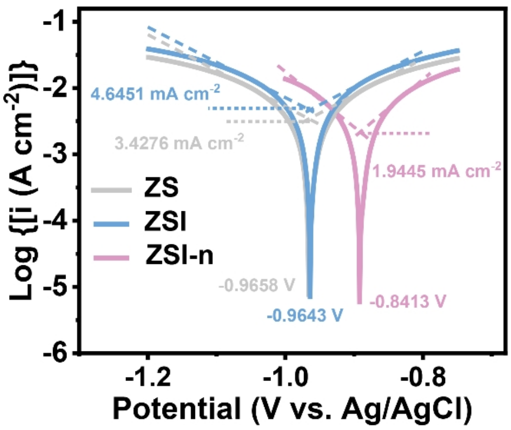

Supplementary Fig. 25 Tafel curves of ZS, ZSI and ZSI-*n* electrolytes.

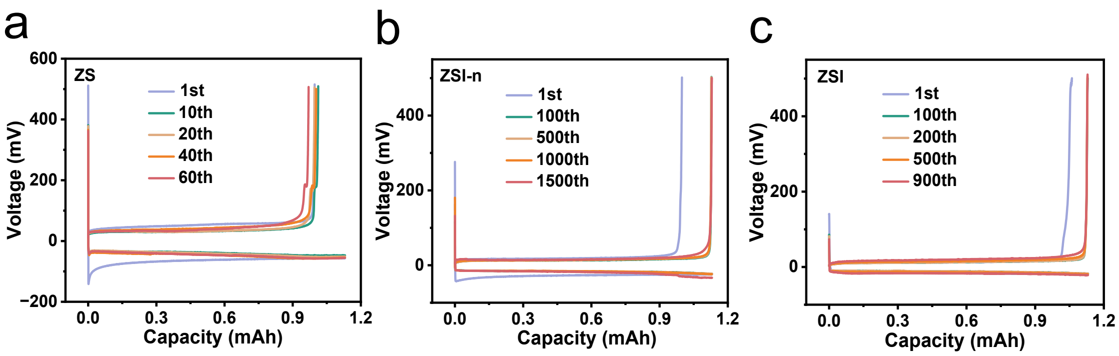

Supplementary Fig. 26 Voltage profiles of Zn//Cu cells using (a) ZS, (b) ZSI and (c) ZSI-*n* electrolyte.

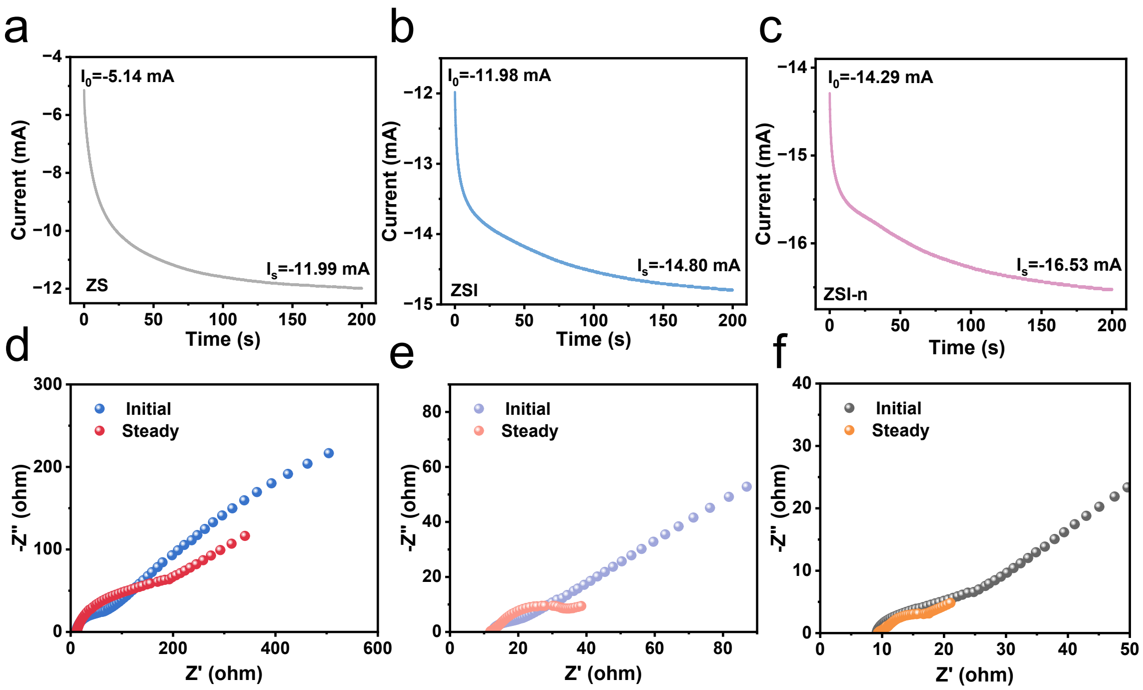

Supplementary Fig. 27 CA curve of the symmetric cell in (a) ZS, (b) ZSI and (c) ZSI-*n* electrolytes and the corresponding EIS plots (d) ZS, (e) ZSI and (f) ZSI-*n* (The corresponding equivalent circuit is consistent with Supplementary Fig. 11)

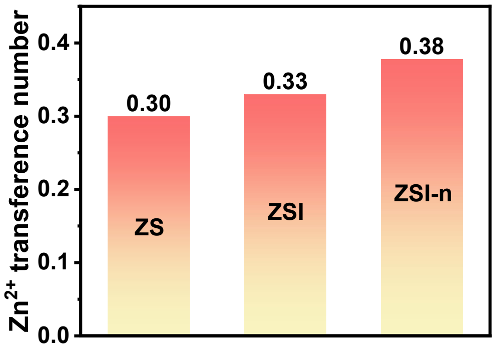

Supplementary Fig. 28 Zn^{2+} transference number (t_{Zn2+}) of the symmetric cell with ZS, ZSI and ZSI-*n* electrolytes.

The transference number of different electrolytes is obtained using the following formula:

$$t_{Zn^{2+}} = \frac{I_s(\Delta V - R_0)}{I_0(\Delta V - R_s)}$$

Where ΔV represents the applied voltage range, I_0 and R_0 denote the initial state current and resistance, while I_s and R_s stand for the steady-state current and resistance.

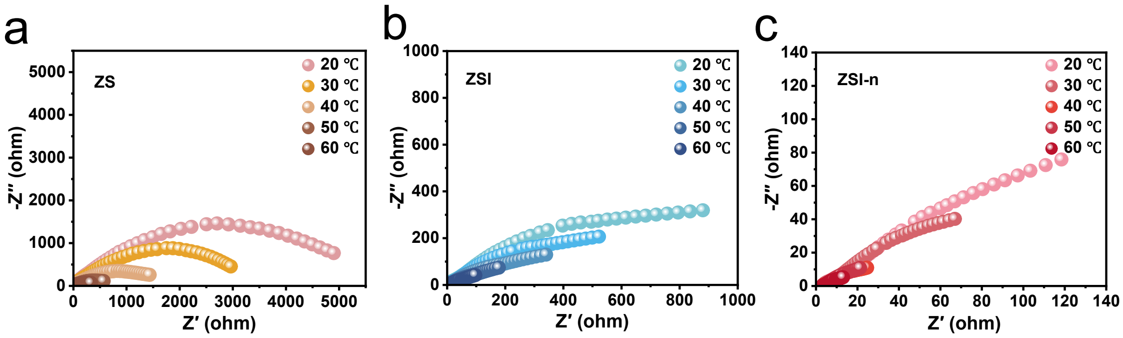

Supplementary Fig. 29 The EIS impedance of Zn//Zn battery in (a) ZS, (b) ZSI and (c) ZSI-*n* electrolytes at different temperature. (The corresponding equivalent circuit is consistent with Supplementary Fig. 11)

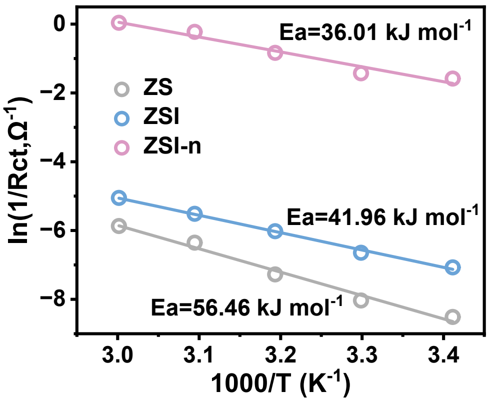

Supplementary Fig. 30 Charge transfer resistances for varying temperature of ZS, ZSI and ZSI-*n* electrolytes.

the activation energy (E_a) can be estimated based on the charge-transfer resistances at different temperatures, according to the Arrhenius equation:

$$\frac{1}{R_{ct}} = Aexp\left(\frac{-E_a}{RT}\right)$$

Where A represents the pre-exponential factor, R is the gas constant, and T stands for temperature.

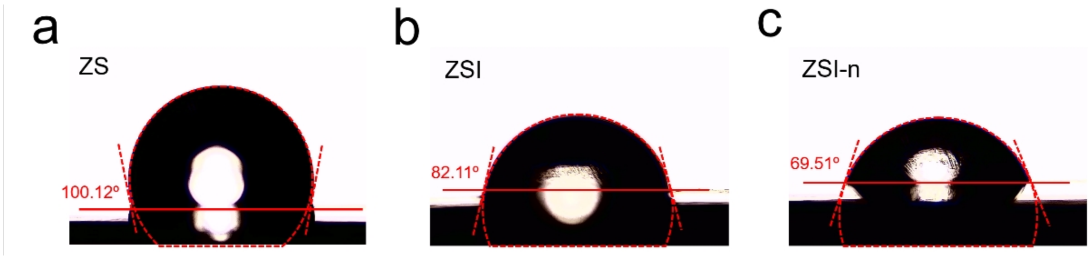

Supplementary Fig. 31 The contact angle of (a) ZS, (b) ZSI and (c) ZSI-*n* electrolytes on bare Zn.

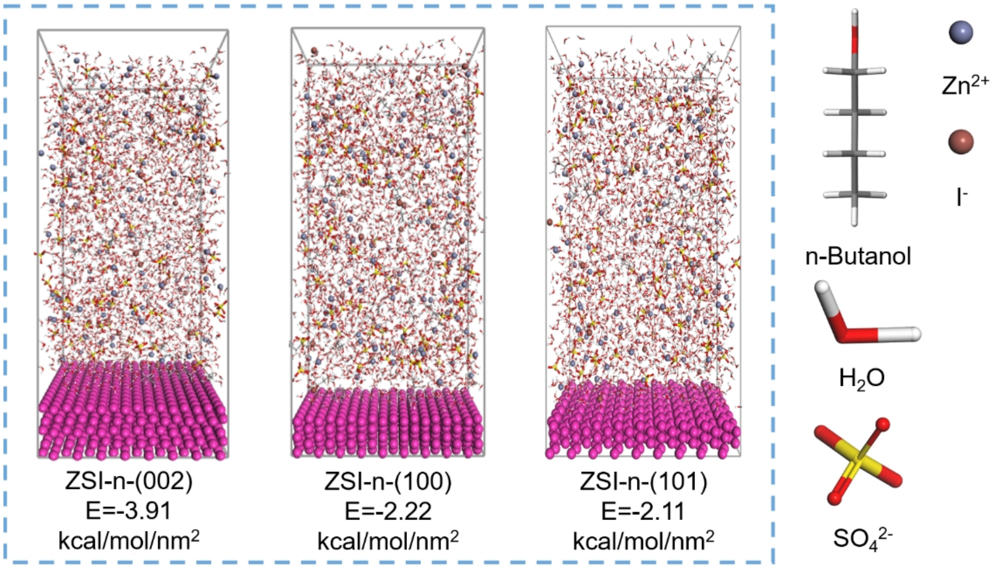

Supplementary Fig. 32 Surface energy for (002), (100) and (101) plane of metallic Zn when exposed in ZS electrolyte.

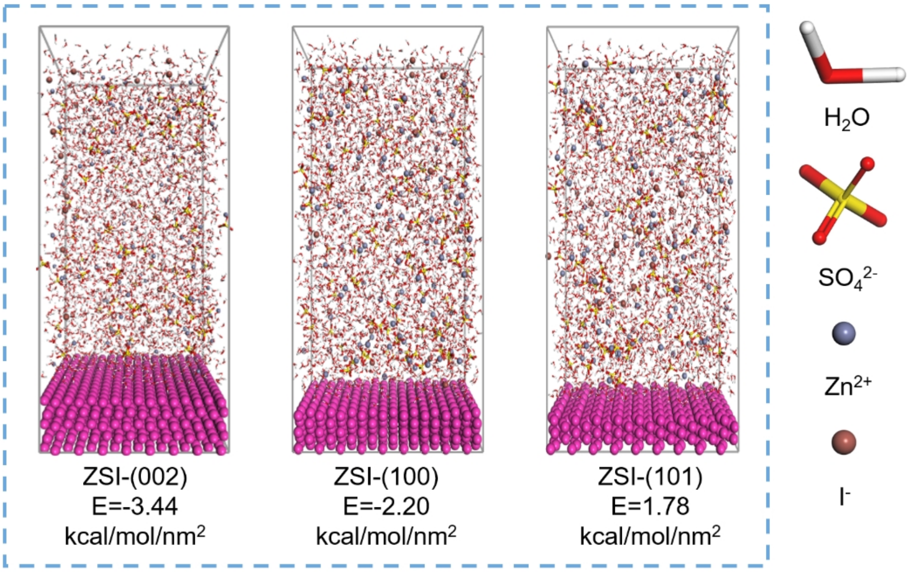

Supplementary Fig. 33 Surface energy for (002), (100) and (101) plane of metallic Zn when exposed in ZSI electrolyte.

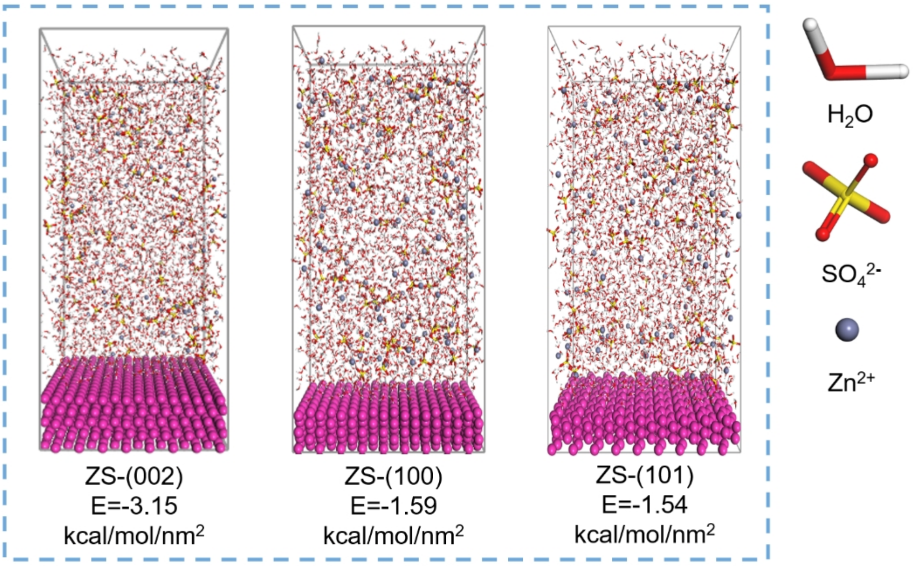

Supplementary Fig. 34 Surface energy for (002), (100) and (101) plane of metallic Zn when exposed in ZSI-*n* electrolyte.

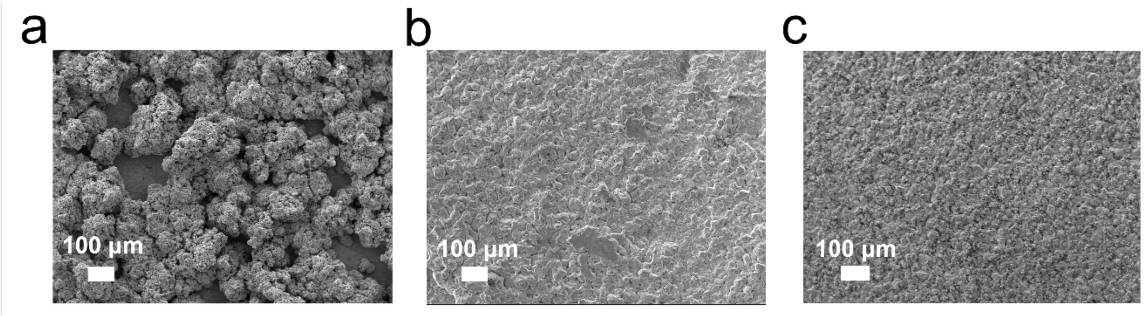

Supplementary Fig. 35 SEM images of Zn anode after 50 cycled in the (a) ZS, (b) ZSI and (c) ZSI-*n* electrolytes.

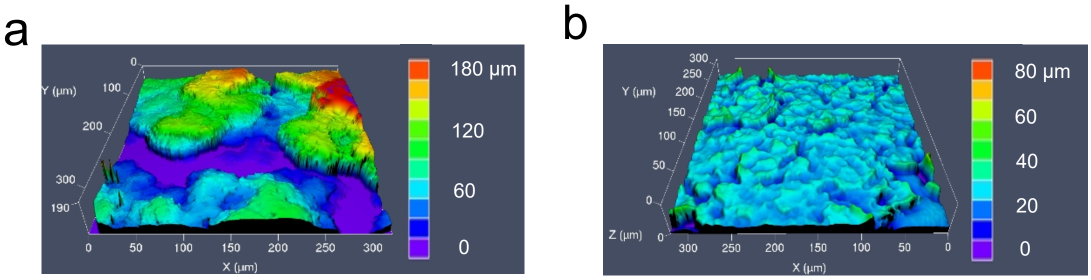

Supplementary Fig. 36 LCSM images of Zn anodes after cycling in (a) ZS and (b) ZSI electrolytes.

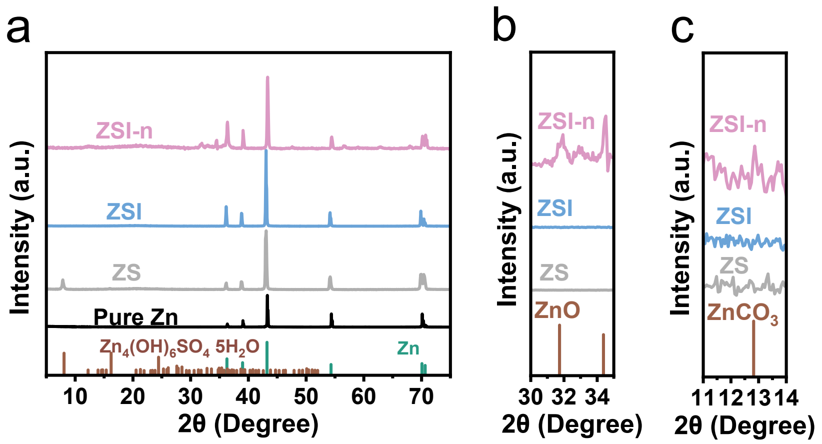

Supplementary Fig. 37 (a) XRD patterns of the electrodes in Zn//Zn symmetrical cells after 50 cycles at 1 mA cm^{-2} and 1 mAh cm^{-2} in ZS, ZSI and ZSI-*n*. The corresponding magnified XRD patterns at (b) 30-35º and (c) 11-14º for three electrolytes systems

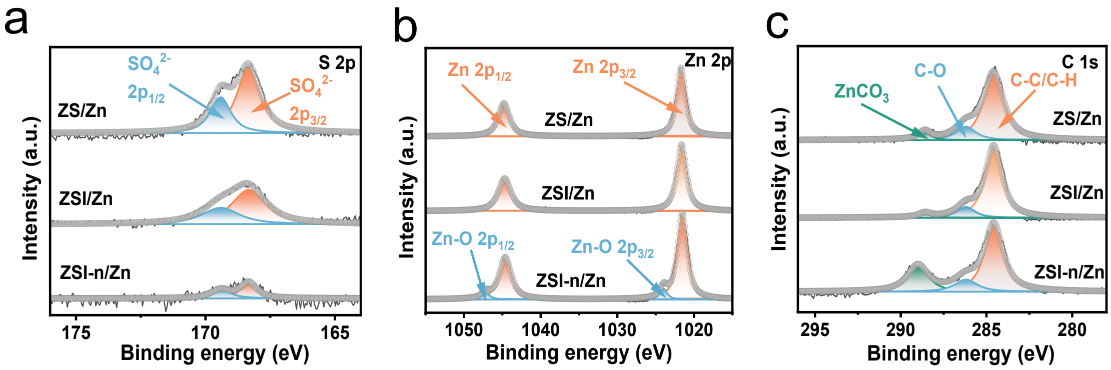

Supplementary Fig. 38 XPS patterns of the electrodes in Zn//Zn symmetrical cells after 50 cycles at 1 mA cm^{-2} and 1 mAh cm^{-2} in ZS, ZSI and ZSI-*n* for (a) S 2p; (b) Zn 2p; (c) C 1s

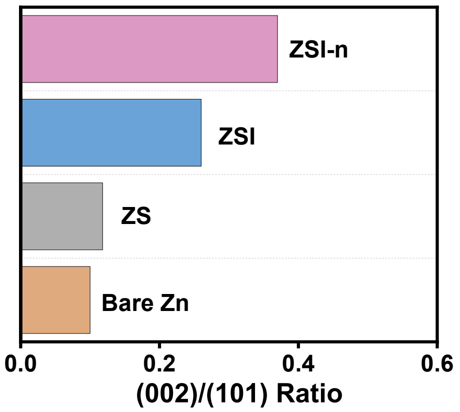

Supplementary Fig. 39 The ratio of (002)/(101) calculated from XRD pattern in Supplementary Fig. 37a.

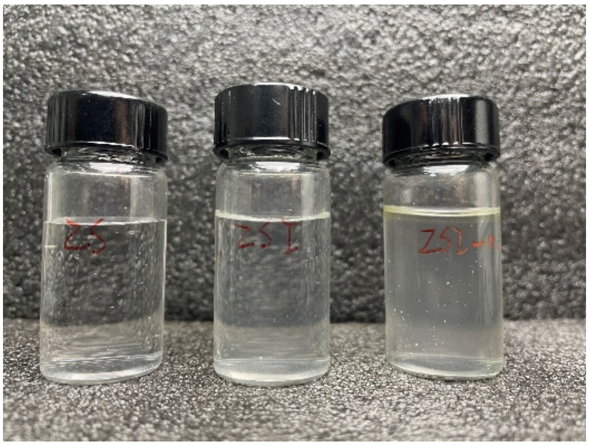

Supplementary Fig. 40 The pictures of ZS (the left), ZSI (the middle) and ZSI-*n* (the right) electrolytes.

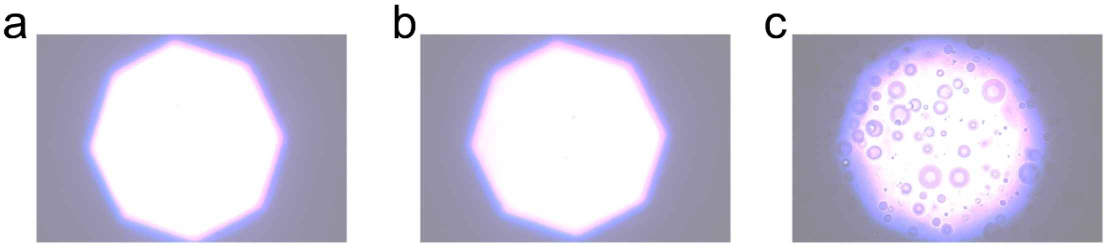

Supplementary Fig. 41 Optical microscopy images of (a) ZS, (b) ZSI and (c) ZSI-*n* electrolytes.

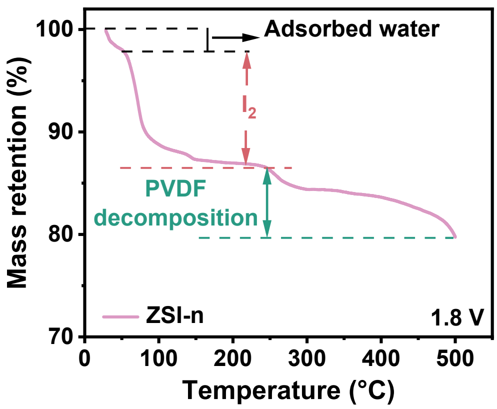

Supplementary Fig. 42 TG curve of the cathode electrode in ZSI-*n*.

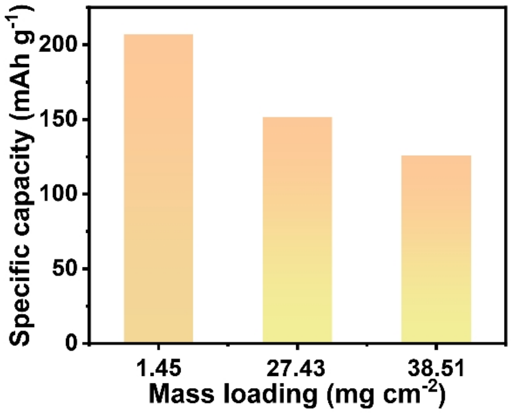

Supplementary Fig. 43 Performance of specific capacity with different mass loading.

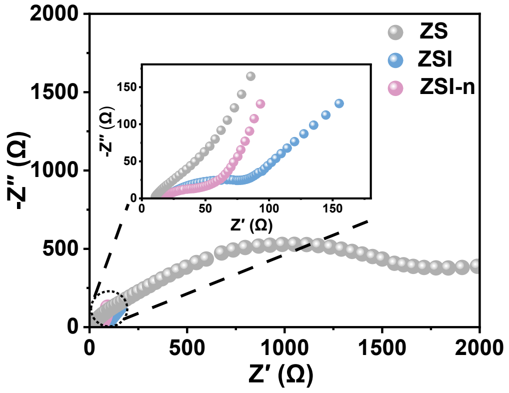

Supplementary Fig. 44 EIS curves of ZS, ZSI and ZSI-*n*. (The corresponding equivalent circuit is consistent with Supplementary Fig. 11)

Supplementary Table 1. Summary of Zn//Zn symmetrical testing performance and function mechanism of alcohol electrolyte additives.

Reference	Electrolyte	Specific current (mA cm^{-2})	Areal capacity (mAh cm^{-2})	Lifespan (h)	Function mechanism
[1]	$ZnSO_4$+ EG	2	1	150	Electrolyte optimization
[2]	$Zn(TFSI)_2$+LiTFSI +PEG	0.6	0.1	450	Electrolyte optimization; Surface film adjustment
[3]	NaTfO+$Zn(TfO)_2$+ ethanol	0.5	0.5	650	Electrolyte optimization; Surface film adjustment
[4]	$ZnSO_4$+methanol	1	0.5	900	Electrolyte optimization
[5]	$ZnSO_4$+sorbitol	1	1	1000	Electrolyte optimization; Orientation adjustment
[6]	$ZnSO_4$+ PG	2	2	1000	Electrolyte optimization; Orientation adjustment
[7]	$ZnSO_4$+tripropylene glycol	1	0.2	1100	Electrolyte optimization; Orientation adjustment
[8]	$ZnSO_4$+ EG	0.5	0.5	1300	Electrolyte optimization; Orientation adjustment
[9]	$Zn(CF_3SO_3)_2$ +PEG	0.25	0.125	1500	Electrolyte optimization; Orientation adjustment
[10]	$ZnSO_4$+ inositol	1	1	1700	Electrolyte optimization; Orientation adjustment
[11]	$ZnSO_4$+sorbitol	1	0.5	2000	Electrolyte optimization; Surface adjustment
**This work**	**$ZnSO_4$+ZnI_2+ *n*-butanol**	**1**	**1**	**3000**	**Electrolyte optimization; Orientation adjustment; Surface film adjustment**

Supplementary Table 2. Summary of the optimization strategy, load mass, specific current, area capacity density, area energy density and cycle number of Zn-I_2 batteries reported recently.

Reference	Optimization strategy	Load mass (mg cm^{-2})	Specific current (A g^{-1})	Area capacity density (mAh cm^{-2})	Area energy density (mWh cm^{-2})	Cycle number
[12]	Cathode	0.06	1	0.014	0.016	1500
[13]	Cathode<br>Anode	1.0	2.5	0.118	0.13	3000
[14]	Cathode	1.0	2.11	0.175	0.21	10000
[15]	Anode	0.63	2	0.126	0.15	10000
[16]	Electrolyte additive<br>*In-situ* SEI	1.38	1.45	0.259	0.30	5000
[17]	Cathode	1.0	5	0.095	0.11	20000
[18]	Anode	1.3	2	0.135	0.15	20000
[19]	Anode	1.0-2.0	3.2	0.09-0.17	0.10-0.20	6000
[20]	Cathode	1.0	1	0.2	0.25	5700
[21]	Cathode	1.0	4.22	0.14	0.16	10000
[22]	Cathode	0.8-1.5	2	0.16-0.3	0.19-0.35	10000
[23]	Cathode	1.25	4	0.25	0.28	1500
[24]	Gel electrolyte	1.5	1.58	0.24	0.26	5000
[25]	Cathode	2	5	0.24	0.29	5000
[26]	Cathode	2	8.44	0.16	0.19	6000
[27]	Cathode	1.0-1.2	2.11	0.15-0.18	0.16-0.20	20000
[28]	Cathode	1.17	4.22	0.154	0.20	5000
[29]	Cathode	1.0-1.2	1.2	0.21-0.25	0.27-0.32	1000
[30]	Cathode	1.0-1.2	5	0.16-0.19	0.19-0.22	30000
[31]	Gel electrolyte	0.8-1.0	2.11	0.12-0.15	0.14-0.17	22000
[32]	Ionic liquid electrolyte	0.8-1.0	4	0.12-0.15	0.14-0.17	18000
[33]	Cathode	1.0-1.2	5	0.17-0.2	0.20-0.24	17000
[34]	Cathode	1.0-2.0	2	0.1-0.2	0.12-0.23	1400
[35]	Cathode	0.8-1.2	3	0.1-0.15	0.12-0.18	5900
**This work**	**Cathode<br>Electrolyte additive<br>*In-situ* SEI**	**1.45**	**5**	**0.30**	**0.34**	**30000**

Supplementary Table 3. Number of electrolyte compositions

Label	$ZnSO_4$	ZnI_2	H_2O	Butanol
S1	100	0	2750	0
S2	100	10	2750	0
S3	100	10	2750	21

Note 1: The mechanism of iodine conversion reaction on TMX is further analyzed in this study. First, *ex-situ* UV test on the electrolyte in the cathode region is conducted, and the results are shown in the Supplementary Fig. 4a. During the charging process, the concentration of I_3^- in the electrolyte near the cathode region continues to increase, indicating that I_2 continues to be generated on the cathode electrode and combines with I^- to form I_3^-[36]. The peak located at 113 cm^{-1} in Raman testing also continues to increase during the charging process (Supplementary Fig. 5a), indicating an increase of I_3^-, which is consistent with the UV results. Moreover, XPS test is conducted on the cathode (Supplementary Fig. 6), and it is found that the peak intensities of I_3^- located at 632.36 and 620.54 eV continue to increase during the charging process, indicating the continuous generation of I_3^-, also consistent with the UV test results. The peaks of I_2 (631.87 and 620.15 eV) increase during the charging process, while the peaks of I^- (631.13 and 619.64 eV) decrease, indicating that I^- ions are continuously converted into I_2 and loaded on the cathode during the charging process[15,20]. XRD test is further conducted on the cathode under different charge states (Supplementary Fig. 7). Compared with the initial cathode, the fully discharged cathode shows significant peak enhancement at 25.50° and 42.50°, which corresponding to ZnI_2 (PDF#10-0072). Besides, there is no obvious I_2 peak, indicating that most of the I_2 is reduced to I^- ions in the fully discharged state[25]. When fully charged to 1.8V, the peak at 25.50° is almost indistinguishable, while a clear peak corresponding to I_2 appears at 24.47° (PDF#43-0304), indicating that most I^- ions are oxidized to I_2 during the charging process. In summary, the following conversion reactions occur on the cathode during the charging and discharging process. During charging, the electrons of I^- are converted into I_2, which combines with I^- to form I_3^-. During discharging, I_2 or I_3^- lose electrons and convert into I^-.

Discharging: $I_2/I_3^- + 2e^- \rightarrow 2I^-/3I^-$

Charging: $2I^-/3I^- - 2e^- \rightarrow I_2/I_3^-$

Note 2: Three electrolytes with different contents of ZnI_2 (2 M $ZnSO_4$+0.2 M ZnI_2, 2 M $ZnSO_4$+0.5 M ZnI_2 and 2 M $ZnSO_4$+1.0 M ZnI_2) were prepared for long cycle testing and Zn//Zn symmetric testing. From the Supplementary Fig. 8, it can be seen that electrolytes containing different concentrations of ZnI_2 have little effect on capacity, and even the capacity decreases in electrolytes containing high concentrations of ZnI_2. Besides, the battery with an electrolyte containing 0.2 M ZnI_2 exhibits a high Coulombic efficiency of 93%, which is much higher than the other two electrolytes (0.5 M ZnI_2: 12%; 1.0 M ZnI_2: 2%). This is because high concentrations of I^- are more likely to combine with I_2 to form I_3^-, leading to overcharging and the production of more I_3^-, resulting in severe shuttle effects. In addition, the effect of different concentrations of ZnI_2 on the zinc anode is further investigated. As shown in the Supplementary Fig. 9, symmetric batteries with high concentrations of ZnI_2 exhibit greater polarization voltage and lower charge discharge efficiency. On the other hand, the lower ion concentrations than 0.2 M will affect the mass loading of I_2 and capacity of the battery. Therefore, 0.2 M is selected as the optimal concentration for subsequent experiments.

Note 3: To find the optimal amount, different concentration of n-butanol (1% v/v, 3% v/v, 6% v/v) is incorporated into the ZSI electrolyte, which are named as ZSI-*n*-1%, ZSI-*n*-3%, and ZSI-*n*-6%, respectively. As shown in Supplementary Fig. 13, the full cell with the ZSI-*n*-3% electrolyte exhibits a significantly improved specific capacity, reaching 0.47 mAh cm^{-2} at 0.5 A g^{-1}. At the same specific current, the specific capacities of cells with ZSI-*n*-1% and ZSI-*n*-6% are 0.34 mAh cm^{-2} and 0.45 mAh cm^{-2}, respectively, both lower than that of ZSI-*n*-3%. Furthermore, at a high specific current of 10 A g^{-1}, the full cell with ZSI-*n*-3% maintains a specific capacity of 0.30 mAh cm^{-2}, significantly higher than those with ZSI-*n*-1% (0.03 mAh cm^{-2}) and ZSI-*n*-6% (0.26 mAh cm^{-2}).

More importantly, the Zn-I_2 battery with ZSI-*n*-3% demonstrates excellent reversible capacity of 0.30 mAh cm^{-2} after 30,000 cycles at a high specific current of 5 A g^{-1}, with a low capacity decay rate of approximately 0.0012% per cycle (Supplementary Fig. 14). In contrast, the battery with ZSI-*n*-1% begins to decay immediately, likely due to insufficient *n*-butanol to limit the I_3^- shuttle effect, resulting in significant iodine loss (as shown in the left image of Supplementary Fig. 15, where the I_3^- shuttle effect is not fully suppressed). Conversely, the battery with ZSI-*n*-6% initially maintains good stability over the first 3,500 cycles with minimal capacity degradation, likely due to the high amount of *n*-butanol effectively fixing I_3^- near the cathode. However, in subsequent cycles, the battery shows significant performance decline, with capacity approaching zero by the end of 10,000 cycles. This decline may be attributed to excessive *n*-butanol accumulation on the cathode surface, which impedes the participation of the outer layer of I_3^- in the reaction, leading to instability in the conversion reaction (evident from the right image of Supplementary Fig. 15, where I_3^- is entirely fixed at the cathode electrode by *n*-butanol).

In summary, too little *n*-butanol fails to adequately mitigate the I_3^- shuttle effect, while excessive *n*-butanol hinders I_3^- participation in the reaction. Therefore, a moderate concentration of *n*-butanol ensures efficient conversion reactions while minimizing iodine loss, which is why we selected 3% v/v as the optimal amount.

Note 4: Thermodynamically, the I_2 is able to combine with I^- ions to form I_3^- ions[37,38]. Thus, the continuous production of I_3^- ions in the electrolyte near the cathode creates a concentration gradient, promoting the diffusion of I_3^- towards the anode. As shown in Supplementary Fig. 4, when charged to 1.8V for the first time (the red line), a large amount of I_3^- ions are generated in the cathode region electrolyte, with a small amount also appearing in the anode region. This indicates that I_3^- ions move from the cathode to the anode during the charging process due to the concentration gradient. During the discharging process, under the influence of an external potential, the anions in the electrolyte move to the anode. As shown by the red and gray curves in Supplementary Fig. 4, the concentration of I_3^- ions in the anode region gradually increases during the discharging process. In the subsequent charging process, I_3^- ions move back to the cathode region due to the electric field. From the Raman spectrum (Supplementary Fig. 5), it can also be seen that in the fully charged state, the peak of I_3^- at 113 cm^{-1} is highest in the cathode region and lowest in the anode region. While in the fully discharged state, the peak of I_3^- is lowest in the cathode region and highest in the anode region[20], which is consistent with the UV results. Therefore, the mechanism of the I_3^- ions shuttle involves its movement from the anode to the cathode during charging and from the cathode to the anode during discharging, similar with the previous reports[18].

Note 5: The inhibitory effect of n-butanol on I_3^- ions shuttle is further confirmed by experiments. Firstly, the photos of ZSI and ZSI-*n* systems are recorded when they are fully charged (Supplementary Fig. 19). The yellow substance (I_3^- ions) produced in the cathode region of the ZSI-*n* system is significantly less than that in the ZSI system, indicating that the addition of *n*-butanol reduces the production of I_3^- ions. Additionally, the anode region of the ZSI-*n* system is colorless, while the anode region of the ZSI system has a distinct yellow color, indicating that *n*-butanol can effectively fixe I_3^- ions in the cathode region, limiting its shuttle to the anode.

Ex-situ UV and Raman tests are further conducted on the electrolytes in the cathode and anode regions, respectively. For the ZSI electrolyte without the *n*-butanol additive (blue curve in Supplementary Fig. 20), the content of I_3^- ions in the electrolyte in the cathode region continues to increase during charging process (Supplementary Figs. 20a-c), indicating the generation I_3^- ions. However, as shown in Supplementary Figs. 20d-f, the content of I_3^- ions in the anode region electrolyte decreases as the charging process progresses, indicating that I_3^- ions are moving back from the anode region to the cathode region. From the Raman spectrum (Supplementary Fig. 5), it can also be seen that in the fully charged state, the peak of I_3^- ions at 113 cm^{-1} is highest in the cathode region and lowest in the anode region. While in the fully discharged state, the peak of I_3^- ions is lowest in the cathode region and highest in the anode region[9], which is consistent with the UV results.

After adding the *n*-butanol additive, as shown in the UV spectrum (Supplementary Fig. 20), there is no significant production of I_3^- ions in the cathode region during continuous charging. Besides, in the Raman spectrum (Supplementary Fig. 21), it is found that at different charging and discharging stages and electrode regions, the peaks of I_3^- ions (113 cm^{-1}) with *n*-butanol added are lower than that without *n*-butanol, indicating that the *n*-butanol can inhibit the formation of I_3^- ions. Additionally, there is no significant change in the concentration of I_3^- ions in the anode region, indicating that *n*-butanol can prevent the shuttle of I_3^- ions.

Note 6: Unlike most reports, where I_2 is loaded directly on the cathodes prior to battery assembly, the I_2 is *in-situ* electrodeposited on cathode from I^- ions during charging process in this study. Here, the mass loading of I_2 on the cathode can be analyzed through TG test[39]. As shown in the Supplementary Fig. 43, the initial weight loss is caused by the loss of adsorbed water, and the subsequent weight loss until 247 °C is due to the loss of I_2, with an I_2 content of 1.45 mg cm^{-2}. In addition, the sustained weight loss from 247 °C to 500 °C is the thermal decomposition of PVDF[40,41].

Supplementary References

1. Chang, N. et al. An aqueous hybrid electrolyte for low-temperature zinc-based energy storage devices. *Energy Environ. Sci.* **13,** 3527-3535 (2020).

2. Lu, H. et al. Manipulating Zn^{2+} solvation environment in poly(propylene glycol)-based aqueous Li^{+}/Zn^{2+} electrolytes for high-voltage hybrid ion batteries. Carbon Energy. **5,** e365 (2023).

3. Sun, Y. et al. Low-cost and long-life Zn/Prussian blue battery using a water-in-ethanol electrolyte with a normal salt concentration. *Energy Storage Mater.* **48,** 192-204 (2022).

4. Hao, J. et al. Boosting Zinc Electrode Reversibility in Aqueous Electrolytes by Using Low-Cost Antisolvents. *Angew. Chem. Int. Ed.* **60,** 7366-7375 (2021).

5. Qiu, M. et al. Metal-coordination chemistry guiding preferred crystallographic orientation for reversible zinc anode. *Energy Storage Mater.* **49,** 463-470 (2022).

6. Shang, Y. et al. Long-Life Zn Anode Enabled by Low Volume Concentration of a Benign Electrolyte Additive. *Adv. Funct. Mater.* **32,** 2200606 (2022).

7. Liu, Z. et al. A Dual-Functional Organic Electrolyte Additive with Regulating Suitable Overpotential for Building Highly Reversible Aqueous Zinc Ion Batteries. *Adv. Funct. Mater.* **34,** 2214538 (2023).

8. Qiu, R. et al. Tuning Zn^{2+} coordination environment to suppress dendrite formation for high-performance Zn-ion batteries. *Nano Energy,* **80,** 105478 (2021).

9. Cao, Z. et al. Ultrastable Zinc Anode by Simultaneously Manipulating Solvation Sheath and Inducing Oriented Deposition with PEG Stability Promoter. *Small,* **18,** 2103345 (2022).

10. Ji, H. et al. Stabilizing zinc anode for high-performance aqueous zinc ion batteries via employing a novel inositol additive. *J. Alloys Compd.* **914,** 165231 (2022).

11. Quan, Y. et al. Electrolyte additive of sorbitol rendering aqueous zinc-ion batteries with dendrite-free behavior and good anti-freezing ability. *Chem. Eng. J.* **458,** 141392 (2023).

12. Wang, S. et al. Halide Exchange in Perovskites Enables Bromine/Iodine Hybrid

Cathodes for Highly Durable Zinc Ion Batteries. *Adv. Mater.* **36,** 2401924 (2024).
13. Yang, J. et al. Janus Binder Chemistry for Synchronous Enhancement of Iodine Species Adsorption and Redox Kinetics toward Sustainable Aqueous Zn-I_2 Batteries. *J. Am. Chem. Soc.* **146,** 6628-6637 (2024).
14. Chai, L. et al. In-MOF-Derived Hierarchically Hollow Carbon Nanostraws for Advanced Zinc-Iodine Batteries. *Adv. Sci.* **9,** 2105063 (2022).
15. Gao, W. et al. Efficient Charge Storage in Zinc-Iodine Batteries based on Pre-Embedded Iodine-Ions with Reduced Electrochemical Reaction Barrier and Suppression of Polyiodide Self-Shuttle Effect. *Adv. Funct. Mater.* **33,** 2211979 (2023).
16. Yan, L. et al. Multifunctional porous carbon strategy assisting high performance aqueous zinc-iodine battery. *Carbon,* **187,** 145-152 (2022).
17. Chen, S. et al. Interface Coordination Stabilizing Reversible Redox of Zinc for High-Performance Zinc-Iodine Batteries. *Small*, **18,** 2200168 (2022).
18. Yang, F. et al. Single atom catalysts for triiodide adsorption and fast conversion for boosted performance in aqueous zinc-iodine batteries. *Energy Environ. Sci.* **16,** 4630-4640 (2023).
19. Guo, C. et al. Cobalt Single-Atom Electrocatalysts Enhanced by Hydrogen-Bonded Organic Frameworks for Long-Lasting Zinc-Iodine Batteries. *Adv. Funct. Mater.* **34,** 2314189 (2024).
20. Wang, S. et al. Conversion-Type Organic-Inorganic Tin-Based Perovskite Cathodes for Durable Aqueous Zinc-Iodine Batteries. *Adv. Energy Mater.* **13,** 2300922 (2023).
21. Qu, W. et al. Ni Single-Atom Bual Catalytic Electrodes for Long Life and High Energy Efficiency Zinc-Iodine Batteries. *Small,* **20,** 2310475 (2024).
22. Sun, J. et al. Heavily heteroatoms doped carbons with tunable microstructure as the iodine hosts for rechargeable zinc-iodine aqueous batteries. J. Alloys Compd. **947,** 169696 (2023).
23. Liu, M. et al. Physicochemical Confinement Effect Enables High-Performing Zinc-Iodine Batteries. *J. Am. Chem. Soc.* **144,** 21683-21691 (2022).
24. Peng, H. et al. Constructing fast-ion-conductive disordered interphase for high-performance zinc-ion and zinc-iodine batteries. *Matter,* **5,** 1-16 (2022).

25. Wei, F. et al. 2D Mesoporous Naphthalene-Based Conductive Heteroarchitectures toward Long-Life, High-Capacity Zinc-Iodine Batteries. *Adv. Funct. Mater.* **34,** 2310693 (2023).

26. Xu, Z, et al. Crowding Effect-Induced Zinc-Enriched/Water-Lean Polymer Interfacial Layer Toward Practical Zn-Iodine Batteries. *ACS Nano*, **17,** 23207-23219 (2023).

27. He, J. et al. Synergistic Effect of Lewis Acid-Base and Coulombic Interactions for High-performance Zn-I_2 Batteries. *Energy Environ. Sci.* **17,** 323-331 (2024).

28. Li, Y. et al. Built-in electrocatalytic nanoreactors anchoring ultrahigh iodine utilization for long-lasting zinc-iodine batteries. *Chemical Engineering Journal,* **483,** 149320 (2024).

29. Xiao, T. et al. All-Round Ionic Liquids for Shuttle-Free Zinc-Iodine Battery. *Angew. Chem. Int. Ed.* **63,** e202318470 (2024).

30. Wei, F. et al. Mesoporous Poly (3,4-ethylenedioxythiophene): Poly (styrenesulfonate) as Efficient Iodine Host for High-Performance Zinc-Iodine Batteries. *ACS Nano*, **17,** 20643-20653 (2023).

31. Wang, G. et al. In situ Construction of Multifunctional Surface Coatings on Zinc Metal for Advanced Aqueous Zinc-Iodine Batteries. *Adv. Energy Mater.* **14,** 2303221 (2024).

32. Chen, Q. et al. Synergic anchoring of Fe_2N nanoclusters on porous carbon to enhance reversible conversion of iodine for high-temperature zinc-iodine battery. *Nano Energy,* **117,** 108897 (2023).

33. Yang, J. et al. Cation-Conduction Dominated Hydrogels for Durable Zinc-Iodine Batteries. *Adv. Mater.* **36,** 2313610 (2024).

34. Du, Y. et al. Lithiation Enhances Electrocatalytic Iodine Conversion and Polyiodide Confinement in Iodine Host for Zinc-Iodine Batteries. *Adv. Funct. Mater.* **33,** 2304811 (2023).

35. Zhang, Y. et al. Unveiling the Role of Cationic Pyridine Sites in Covalent Triazine Framework for Boosting Zinc-Iodine Batteries Performance. *Adv. Mater.* 2403097 (2024).

36. Lyu, Y. et al. Organic pH Buffer for Dendrite - Free and Shuttle - Free Zn - I_2 Batteries. *Angew. Chem. Int. Ed.* **62,** e202303011 (2023).
37. Weng, G. et al. Unlocking the capacity of iodide for high-energy-density zinc/polyiodide and lithium/polyiodide redox flow batteries. *Energy Environ. Sci.* **10,** 735-741 (2017).
38. Lin, D. et al. Prototypical Study of Double-Layered Cathodes for Aqueous Rechargeable Static Zn-I_2 Batteries. *Nano Lett.* **21,** 4129-4135 (2021).
39. Zhang, Z. et al. Development of long lifespan high-energy aqueous organic|| iodine rechargeable batteries. *Nat. Commun.* **13,** 6489 (2022).
40. Wang, M. et al. A facile, environmentally friendly, and low-temperature approach for decomposition of polyvinylidene fluoride from the cathode electrode of spent lithium-ion batteries. *ACS Sustainable Chem. Eng.* **7,** 12799-12806 (2019).
41 Zhu, X. et al. Improved recovery of cathode materials and enhanced lithium selective extraction from spent $LiNi_{0.5}Co_{0.2}Mn_{0.3}O_2$ batteries via $CaCl_2$-assisted microwave roasting. *J. Environ.* **12,** 112037 (2024).
