# Supplementary material for: A tripartite synergistic optimization strategy for zinc-iodine batteries
Source: Nat Commun. 2024 Nov 9;15:9702. doi: 10.1038/s41467-024-53800-6 (PMC11549484; doi:10.1038/s41467-024-53800-6)
Supplement: Supplementary file 2 — Reporting Summary [file 41467_2024_53800_MOESM2_ESM.pdf]

nature portfolio

Corresponding author(s): Zifeng Lin

Last updated by author(s): Oct 10, 2024

Reporting Summary

Nature Portfolio wishes to improve the reproducibility of the work that we publish. This form provides structure for consistency and transparency in reporting. For further information on Nature Portfolio policies, see our Editorial Policies and the Editorial Policy Checklist.

Statistics

For all statistical analyses, confirm that the following items are present in the figure legend, table legend, main text, or Methods section.

n/a	Confirmed	
☒	☐	The exact sample size (n) for each experimental group/condition, given as a discrete number and unit of measurement
☐	☒	A statement on whether measurements were taken from distinct samples or whether the same sample was measured repeatedly
☒	☐	The statistical test(s) used AND whether they are one- or two-sided<br>*Only common tests should be described solely by name; describe more complex techniques in the Methods section.*
☒	☐	A description of all covariates tested
☒	☐	A description of any assumptions or corrections, such as tests of normality and adjustment for multiple comparisons
☒	☐	A full description of the statistical parameters including central tendency (e.g. means) or other basic estimates (e.g. regression coefficient) AND variation (e.g. standard deviation) or associated estimates of uncertainty (e.g. confidence intervals)
☒	☐	For null hypothesis testing, the test statistic (e.g. F, t, r) with confidence intervals, effect sizes, degrees of freedom and P value noted<br>*Give P values as exact values whenever suitable.*
☒	☐	For Bayesian analysis, information on the choice of priors and Markov chain Monte Carlo settings
☒	☐	For hierarchical and complex designs, identification of the appropriate level for tests and full reporting of outcomes
☒	☐	Estimates of effect sizes (e.g. Cohen's d, Pearson's r), indicating how they were calculated

Our web collection on statistics for biologists contains articles on many of the points above.

Software and code

Policy information about availability of computer code

Data collection	Structural characterizations were conducted on FEI Tecnai F20, Bruker D8 powder X-ray diffractometer, PE lambda 750 UV-Vis spectrophotometer, Thermo Scientific Nicolet iS20 infrared spectroscopy, Horiba LabRAM HR Evolution, Bruker AVANCE 400 spectrometer ; Electrochemical characterizations were conducted on CHI760 electrochemical workstation (Shanghai, China), Neware battery test system (Shenzhen, China).
Data analysis	PowerPoint, Origin, Materials Studio, Photoshop

For manuscripts utilizing custom algorithms or software that are central to the research but not yet described in published literature, software must be made available to editors and reviewers. We strongly encourage code deposition in a community repository (e.g. GitHub). See the Nature Portfolio guidelines for submitting code & software for further information.

Data

Policy information about availability of data

All manuscripts must include a data availability statement. This statement should provide the following information, where applicable:

- Accession codes, unique identifiers, or web links for publicly available datasets
- A description of any restrictions on data availability
- For clinical datasets or third party data, please ensure that the statement adheres to our policy

All data that support the findings of this study are provided within the paper and its Supplementary Information. All additional information is available from the corresponding authors upon request. Source data are provided with this paper.

Research involving human participants, their data, or biological material

Policy information about studies with human participants or human data. See also policy information about sex, gender (identity/presentation), and sexual orientation and race, ethnicity and racism.

Reporting on sex and gender	The findings apply to all sex and gender. No sex and gender were considered in study design.
Reporting on race, ethnicity, or other socially relevant groupings	This is not relevant to our study.
Population characteristics	Not applicable
Recruitment	Not applicable
Ethics oversight	Sichuan university

Note that full information on the approval of the study protocol must also be provided in the manuscript.

Field-specific reporting

Please select the one below that is the best fit for your research. If you are not sure, read the appropriate sections before making your selection.

☐ Life sciences ☐ Behavioural & social sciences ☒ Ecological, evolutionary & environmental sciences

For a reference copy of the document with all sections, see nature.com/documents/nr-reporting-summary-flat.pdf

Ecological, evolutionary & environmental sciences study design

All studies must disclose on these points even when the disclosure is negative.

Study description	A tripartite synergistic optimization strategy is proposed, involving cathode host, electrolyte additive and in-situ anode protection, which enables the zinc-iodine batteries exhibit high capacity, superior energy density, and ultralong cycle life.
Research sample	zinc-iodine batteries, MXene cathode host, n-butanol electrolyte additive, solid electrolyte interface
Sampling strategy	The MXene suspension was dried at 60 ? for 12 h to obtain MXene power. The electrode was prepared by mixing MXene power, Ketjen black and PVDF in a mass ratio of 7:2:1 with the adding of NMP, and then cost the slurry on carbon cloth. After drying at 60 ? for 12 h in a vacuum oven, the carbon cloth covered by mixture was cut into a disc (ϕ = 12 mm) to be used as the cathode electrode. The mass loading of MXene on each disc was about 1 mg/cm2. Based on such cathode, zinc-iodine batteries were assembled by using 2 M ZnSO4 + 0.2 M ZnI2+ 3% (v/v) n-butanol as electrolyte, glass-fibre separator as separator and Zn foil as anode. The thickness, diameter, area, mass loading and active material of cathode electrode are 0.4 mm, 12 mm, 1.13 cm-2, 1.45 mg cm-2 and 70 wt% respectively. The thickness and area of the anode zinc electrode are 0.03 mm and 1.13 cm-2 respectively. The thickness, diameter and area of the glass-fibre separator is 1 mm, 16 mm and 2.01 cm-2, and the amount of electrolyte droplets added to each coin cell is 80 μL.
Data collection	Structural characterizations were conducted on FEI Tecnai F20, Bruker D8 powder X-ray diffractometer, PE lambda 750 UV-Vis spectrophotometer, Thermo Scientific Nicolet iS20 infrared spectroscopy, Horiba LabRAM HR Evolution, Bruker AVANCE 400 spectrometer ; Electrochemical characterizations were conducted on CHI760 electrochemical workstation (Shanghai, China), Neware battery test system (Shenzhen, China).
Timing and spatial scale	Apr. 2023 - Mar. 2024
Data exclusions	No data were excluded.

Reproducibility	All attempts to repeat the experiments are successful.
Randomization	Not applicable
Blinding	Not applicable

Did the study involve field work? ☐ Yes ☒ No

Reporting for specific materials, systems and methods

We require information from authors about some types of materials, experimental systems and methods used in many studies. Here, indicate whether each material, system or method listed is relevant to your study. If you are not sure if a list item applies to your research, read the appropriate section before selecting a response.

Materials & experimental systems

n/a	Involved in the study
☒	☐ Antibodies
☒	☐ Eukaryotic cell lines
☒	☐ Palaeontology and archaeology
☒	☐ Animals and other organisms
☒	☐ Clinical data
☒	☐ Dual use research of concern
☒	☐ Plants

Methods

n/a	Involved in the study
☒	☐ ChIP-seq
☒	☐ Flow cytometry
☒	☐ MRI-based neuroimaging

Plants

Seed stocks	Not applicable
Novel plant genotypes	Not applicable
Authentication	Not applicable
